# Exogenous Application of dsRNA—Inducing Silencing of the *Fusarium oxysporum Tup1* Gene and Reducing Its Virulence

**DOI:** 10.3390/ijms251910286

**Published:** 2024-09-24

**Authors:** Sen Fan, Yanguang Zhou, Na Zhu, Qingling Meng, Yujin Zhao, Jingyan Xu, Yunjia Tang, Shijie Dai, Xiaofeng Yuan

**Affiliations:** 1 School of Life Sciences, Zhejiang Chinese Medical University, Hangzhou 310051, China; fansen2001@163.com (S.F.); zhouyanguang07@163.com (Y.Z.); zhuna85@163.com (N.Z.); 15192866713@163.com (Q.M.); zhaoyujin326@163.com (Y.Z.); 15824357597@163.com (J.X.); tangyunjia@zju.edu.cn (Y.T.); dsj2513140@163.com (S.D.); 2 Future Health Laboratory, Innovation Center of Yangtze River Delta, Zhejiang University, Jiaxing 314102, China

**Keywords:** *Fusarium oxysporum*, *Atractylodes macrocephala*, *Tup1*, spray-induced gene silencing, double-stranded RNA (dsRNA), RNA interference

## Abstract

*Fusarium oxysporum* is a widespread soil-borne fungal pathogen that can infect various plants, causing wilt and root rot diseases. The root rot disease of *Atractylodes macrocephala* caused by *F. oxysporum* is among the most serious diseases associated with continuous cropping, significantly hindering its sustainable development. In this study, we aimed to investigate the effect of exogenous application of double-stranded RNA (dsRNA) on silencing the *F. oxysporum Tup1* gene to reduce its virulence and to evaluate its potential application in controlling root rot disease in *A. macrocephala*. The *Tup1* gene was amplified from the *F. oxysporum* genome, and different lengths of *Tup1*-dsRNA were designed and synthesized. The uptake of dsRNA by the fungus was verified using *Tup1*-dsRNA labeled with fluorescein, and in vitro dsRNA treatment experiments were conducted to assess its impact on the growth and virulence of *F. oxysporum*. Additionally, *Tup1*-dsRNA was applied to the roots of *A. macrocephala* to evaluate its effectiveness in controlling root rot disease. The experimental results showed that *F. oxysporum* could effectively uptake exogenously applied *Tup1*-dsRNA, significantly reducing *Tup1* gene expression. All lengths of *Tup1*-dsRNA inhibited fungal growth and caused morphological changes in the fungal hyphae. Further plant experiments and Reverse Transcription Quantitative Polymerase Chain Reaction (RT-qPCR) analysis indicated that *Tup1*-dsRNA treatment significantly reduced the incidence of root rot disease in *A. macrocephala*, which was supported by the reduction in peroxidase (POD) and catalase (CAT) enzyme activities, malondialdehyde (MDA) content, and proline (Pro) levels in treated root tissues. This study demonstrated that exogenous dsRNA could reduce the virulence of *F. oxysporum* by silencing the *Tup1* gene and effectively mitigate the root rot disease it causes in *A. macrocephala*. The successful application of *Tup1*-dsRNA provided strong evidence for the potential of RNA interference (RNAi) technology in plant disease control. Future research could further optimize the design and application of dsRNA to enhance its practical value in agriculture.

## 1. Introduction

*Fusarium oxysporum* is a ubiquitous soil-borne fungal pathogen that infects various plants, leading to wilt and root rot diseases. It survives in the soil as thick-walled spores and infects plants through root wounds or direct invasion. Once infected, the pathogen spreads through the plant’s vascular system, resulting in leaf yellowing, wilting, and ultimately plant death [1,2].

*Atractylodes macrocephala* is an important medicinal herb primarily used for invigorating the spleen, drying dampness, and stopping sweating [3]. It contains various chemical components, including volatile oils, atractylenolides, polysaccharides, and amino acids, which exhibit pharmacological effects such as anti-inflammatory, anti-tumor, and antiviral activities [4,5]. However, *A. macrocephala* faces replanting obstacles, requiring a 5- to 10-year interval between plantings, which severely limits sustainable development. Among the replanting diseases, root rot caused by *F. oxysporum* is the most severe [6]. The current control methods for *F. oxysporum*-induced root rot include physical rotation and chemical treatments, both of which have limitations. Thus, developing efficient new biological fungicides is crucial for effective biological control.

Small RNA (sRNA) is a type of non-coding RNA found in eukaryotes that is shorter than 200 nucleotides. It plays a role in regulating various biological processes, including plant growth, development, and responses to stress. Additionally, sRNA is being explored as a tool for biological defense, specifically for pest control [7,8]. sRNA is produced by the homologous protein Dicer-like (DCL) and incorporated into Argonaute (AGO) proteins, inducing gene silencing in a sequence-specific manner [9]. As research on sRNA progresses, a regulatory network based on sRNA is emerging. Researchers are developing disease-resistant plants by modifying specific processes within this regulatory network [10], and population control is being actively promoted through artificial sRNA delivery systems, such as host-induced gene silencing (HIGS) and spray-induced gene silencing (SIGS), to reduce pest outbreaks [11]. The gene functions of sRNA provide a promising direction for controlling *A. macrocephala* root rot, with a focus on using SIGS technology.

In recent years, the concept of spray-induced gene-silencing RNA biopesticides has paved the way for developing new biological fungicides [12,13]. Spraying double-stranded RNA (dsRNA) on leaves and soil has been effective in controlling pests [14]. Research has demonstrated that applying dsRNA targeting pathogen virulence factors to plants significantly reduces pest occurrence. For example, RNA spray-mediated gene silencing of *Fusarium graminearum AGO* and *DCL* genes reduced its infection on barley leaves, enhancing barley resistance [15]. Similarly, spraying synthetic *FolRDR1*-dsRNA on tomato plants downregulated *F. oxysporum FolRDR1* gene expression, inhibiting its growth and infection [16]. Additionally, exogenous dsRNA spray-induced *MoDES1* gene silencing enhanced rice resistance to rice blast disease [17]. The exogenous application of *Bmp1*-dsRNA, *Bmp3*-dsRNA, and *Pls1*-dsRNA effectively inhibited *Botrytis cinerea* growth [18,19]. These research findings provide a theoretical basis for developing RNA interference (RNAi) fungicides.

Compared to traditional chemical pesticides and fungicides, RNAi-mediated crop protection strategies are considered highly specific and environmentally friendly, making them a sustainable alternative [11]. RNAi technology can specifically silence key pathogen genes without affecting the normal gene expression of plants and other non-target organisms, offering significant advantages in disease control [20]. Moreover, the dsRNA and Small interfering RNA (siRNA) used in RNAi technology are biological molecules that degrade easily, leaving no long-term residues or causing pollution. Thus, the exogenous application of RNAi is considered a promising strategy to improve crop resistance to diseases and stress [21], while avoiding the ethical and environmental issues associated with transgenics [22].

*Tup1* is a conserved transcriptional corepressor that plays a crucial role in regulating gene expression in response to environmental changes [23]. In *Saccharomyces cerevisiae*, *Tup1* is particularly important for the transcriptional repression observed during quiescence, which is essential for the establishment and maintenance of quiescence, highlighting *Tup1*’s critical role in cellular adaptation to environmental stress [24]. Similarly, in *Candida albicans*, it was found that silencing the *Tup1* gene can affect the expression of key genes associated with fungal virulence, suggesting a potential strategy for fungal control [25]. Recent research on the *F. oxysporum Tup1* gene further underscored its importance, revealing its crucial role in various biological processes and the pathogen’s virulence by regulating gene expression in major metabolic pathways, including the tricarboxylic acid cycle (TCA cycle) [26].

Consequently, we chose the *Tup1* gene as a model to investigate the feasibility of using SIGS to control *A. macrocephala* root rot. In this study, three different lengths of dsRNA targeting the *F. oxysporum Tup1* gene were designed. The silencing effect of exogenous *Tup1*-dsRNA application on the *Tup1* gene was investigated, providing new ideas and insights for the biological control of *A. macrocephala* root rot.

## 2. Results

### 2.1. Identification of Fusarium oxysporum Tup1 Gene

**Figure 1:**
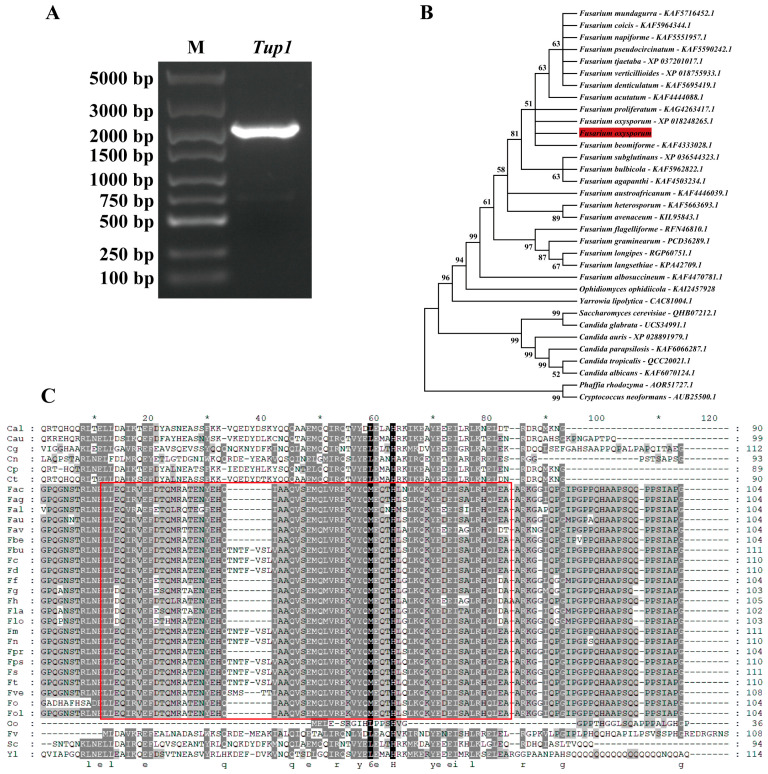
Identification of *F. oxysporum Tup1* gene. (**A**) *Tup1* DNA agarose gel electrophoresis (M: marker); (**B**) Phylogenetic analysis of *Tup1* (the red line represents *F. oxysporum* in this study); (**C**) Amino acid sequence alignment of *Tup1*. Different grey backgrounds in the figure represent specific functional regions or conserved regions in the sequence alignment. Darker backgrounds indicate highly conserved regions, while lighter backgrounds indicate less conserved regions. Red box indicates the Tup_N domain. * marks fully conserved sites. The lowercase letters at the bottom of the figure represent key amino acid residues, “e” stands for glutamic acid, “q” stands for glutamine, “g” stands for glycine, “r” stands for arginine, “y” stands for tyrosine, “l” stands for leucine, and “h” stands for histidine.

The genomic DNA of *F. oxysporum* was used to amplify the *Tup1* gene, and a single band of approximately 2000 bp was obtained through agarose gel electrophoresis (Figure 1A). After sequencing, BLAST analysis (blast.ncbi.nlm.nih.gov/Blast.cgi, Date of access: 16 March 2024) in the NCBI database confirmed it as the *Tup1* gene. The full length of the *Tup1* gene was 1989 bp, containing three introns and encoding 591 amino acids. Structural analysis of the Tup1 protein sequence on the CD SERVER website (www.ncbi.nlm.nih.gov/Structure/cdd/wrpsb.cgi, Date of access: 16 March 2024) showed that the N-terminus contained a 65-amino-acid-long Tup_N domain, and the C-terminus contained six tandem repeats of the WD40 domain. Phylogenetic analysis of the *F. oxysporum* Tup1 protein using the MEGA 11 software (www.megasoftware.net, Date of access: 17 March 2024) indicated that the *Tup1* gene was conserved among *Fusarium* species (Figure 1B). Amino acid alignment using the Clustalw software (http://www.clustal.org, date of access: 17 March 2024) revealed that the Tup_N domain at the N-terminus was highly conserved only within the *Fusarium* species (Figure 1C).

### 2.2. Synthesis of Tup1-dsRNA

**Figure 2:**
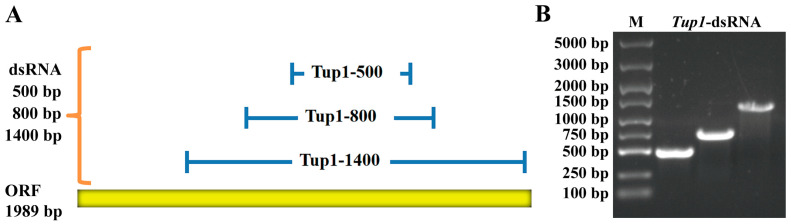
Design and detection of *Tup1*-dsRNA. (**A**) Design of different lengths of *Tup1*-dsRNA; (**B**) Agarose gel electrophoresis results of different lengths of *Tup1*-dsRNA.

Due to the high conservation of the *Tup1* gene in the *Fusarium* species, a basis for designing dsRNA specifically targeting *F. oxysporum* was established. To investigate whether in vitro synthesized *Tup1*-dsRNA could induce silencing of the *F. oxysporum Tup1* gene and affect its normal growth and pathogenicity, three different lengths of *Tup1*-dsRNA targeting the 1989 bp open reading frame of the *Tup1* gene were synthesized: 536 bp dsRNA, 827 bp dsRNA, and 1471 bp dsRNA (referred to as Tup1-500, Tup1-800, and Tup1-1400, respectively) (Figure 2A). To ensure the successful synthesis of the three different lengths of dsRNA in vitro, agarose gel electrophoresis was performed on the synthetic products, and the results confirmed that Tup1-500, Tup1-800, and Tup1-1400 had been successfully synthesized (Figure 2B).

### 2.3. Tup1-dsRNA Was Effectively Taken Up and Absorbed by Fusarium oxysporum

**Figure 3:**
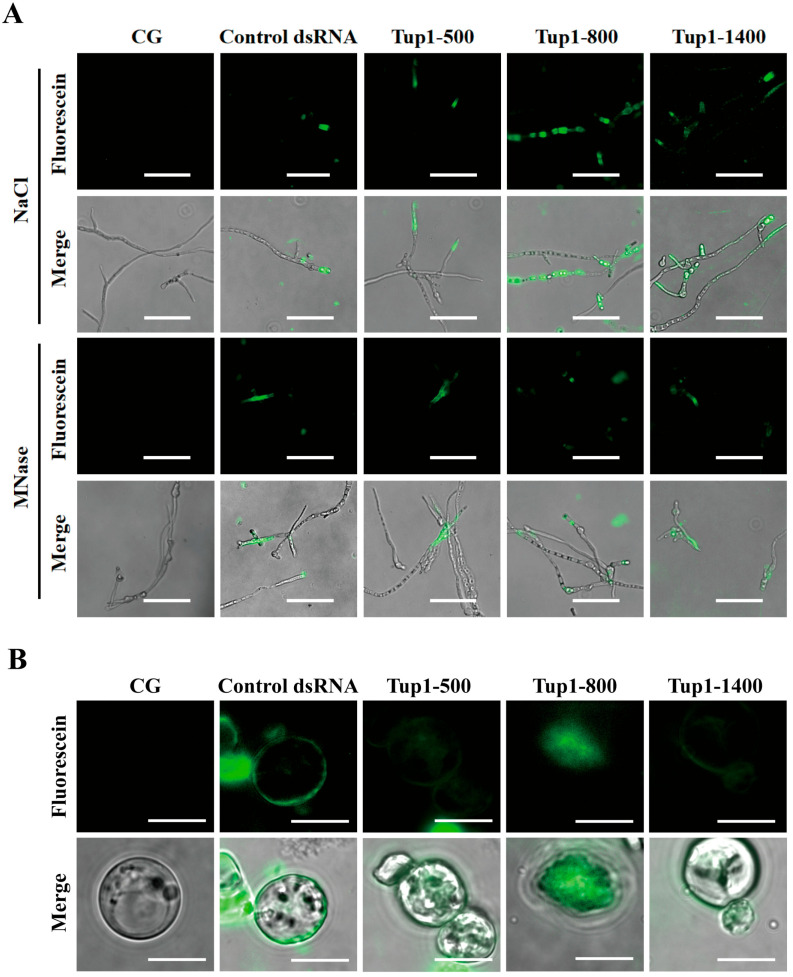
Uptake of *Tup1*-dsRNA by *F. oxysporum*. (**A**) Uptake of fluorescent dsRNA by *F. oxysporum* after co-incubation (MNase: micrococcal nuclease. Scale bar 100 μm); (**B**) Fluorescent dsRNA uptake into *F. oxysporum* protoplasts (Scale bar 10 μm).

To investigate whether dsRNA could be taken up by fungi from the environment, we applied fluorescently labeled *Tup1*-dsRNA to *F. oxysporum* spores. After 24 h, fluorescent signals were observed accumulating inside the fungal cells (Figure 3A). To exclude the possibility that *Tup1*-dsRNA was adhering to the exterior of the fungal cells, the fungi were treated with micrococcal nuclease (MNase), with NaCl used as a control. Fluorescent signals were still detectable, indicating that the dsRNA had been taken up by *F. oxysporum*. Additionally, the control dsRNA, which has no homology to *F. oxysporum*, was also effectively taken up into fungal cells, suggesting that dsRNA uptake is unlikely to be selective. Additionally, to confirm that the dsRNA had entered the cytoplasm of *F. oxysporum* rather than being embedded in the cell wall matrix, we treated *F. oxysporum* protoplasts with fluorescently labeled *Tup1*-dsRNA. The results showed that fluorescent signals were clearly detectable in the protoplasts of *F. oxysporum* (Figure 3B). These indicate that the uptake of environmental *Tup1*-dsRNA by *F. oxysporum* is non-selective.

### 2.4. Effect of Exogenous Application of Tup1-dsRNA on the Growth and Germination of Fusarium oxysporum Spores and Fungal Transcript Analysis

**Figure 4:**
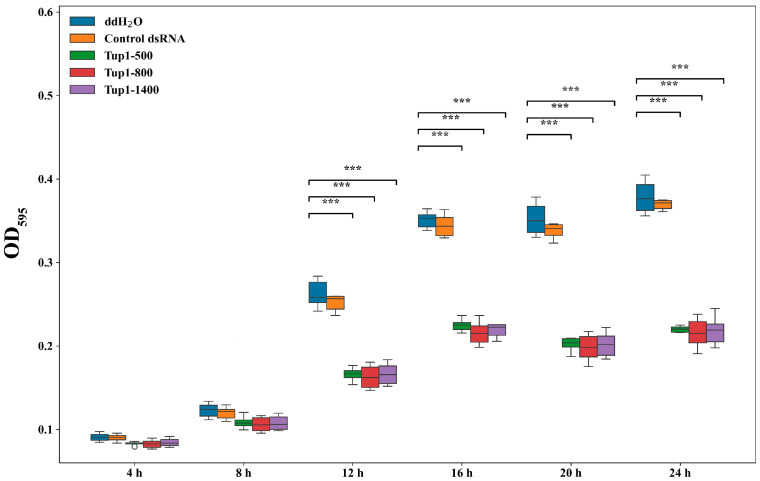
Effect of exogenous application of *Tup1*-dsRNA on the growth of *F. oxysporum* spores (*n* = 6). *** marks *p* < 0.001.

To investigate the effect of the exogenous application of *Tup1*-dsRNA on the growth of *F. oxysporum*, the optical density changes at OD_595_ of fungal spore suspensions in a 96-well plate were measured over 24 h (*n* = 6). After co-culturing Tup1-500, Tup1-800, and Tup1-1400 (50 ng μL^−1^) with *F. oxysporum* spores (1 × 10^6^ cfu mL^−1^), no significant change in the growth of *F. oxysporum* spores treated with control dsRNA was observed compared to the control group over 24 h. After treatment with different lengths of *Tup1*-dsRNA, no significant difference in the growth of *F. oxysporum* spores was observed at 4 h and 8 h. However, after 12 h, the growth of *F. oxysporum* spores treated with *Tup1*-dsRNA began to slow down significantly, and the inhibitory effects of different lengths of *Tup1*-dsRNA on the growth of *F. oxysporum* spores were similar (Figure 4).

**Figure 5:**
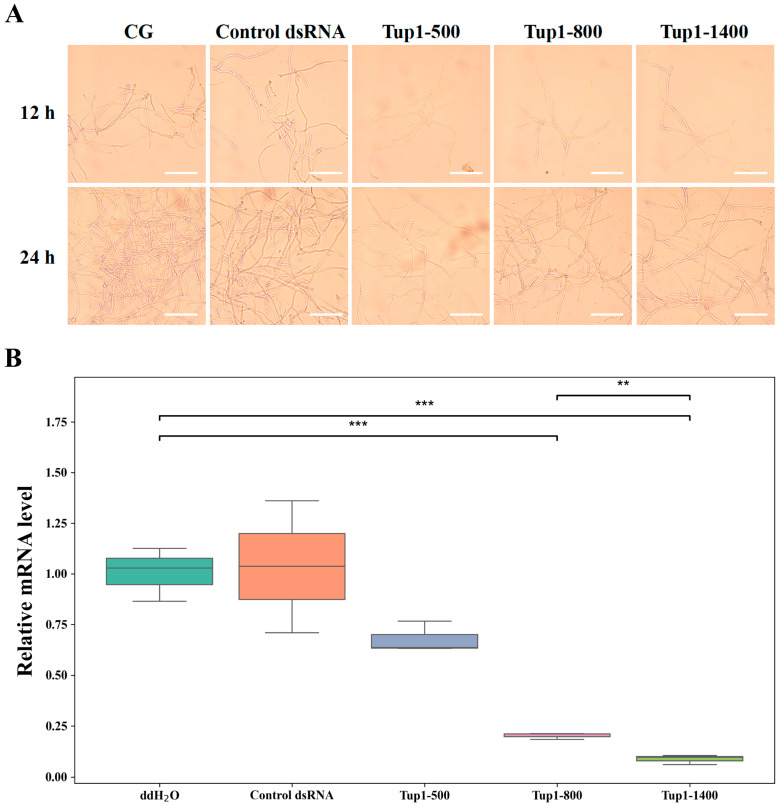
Effect of *Tup1*-dsRNA on the growth morphology of *F. oxysporum* spores. (**A**) Inhibition of *F. oxysporum* growth after co-culturing with *Tup1*-dsRNA (Scale bar: 100 μm); (**B**) Inhibition of *Tup1* gene expression by *Tup1*-dsRNA (*n* = 3). *** marks *p* < 0.001, ** marks *p* < 0.01.

To further investigate the effect of co-culturing *Tup1*-dsRNA with *F. oxysporum* on spore growth morphology and the silencing efficiency of Tup1-500, Tup1-800, and Tup1-1400 on the *Tup1* gene, *F. oxysporum* spore suspensions were collected at different incubation times for microscopic observation and Reverse Transcription Quantitative Polymerase Chain Reaction (RT-qPCR) analysis. The results showed that, in comparison to the control group, spore growth was inhibited and fewer hyphae were observed after treatment with Tup1-500, Tup1-800, and Tup1-1400, with continued inhibitory effects on *F. oxysporum* growth observed at 24 h (Figure 5A). To further evaluate the silencing capability of *Tup1*-dsRNA, RT-qPCR was employed to measure the expression of the *Tup1* gene in *F. oxysporum* after 12 h of treatment with *Tup1*-dsRNA (*n* = 3). The results showed that Tup1-1400 and Tup1-800 significantly reduced the expression of the *Tup1* gene, with Tup1-1400 exhibiting a stronger inhibitory effect than Tup1-800. Meanwhile, Tup1-500 also reduced the expression of the *Tup1* gene, but the difference was not significant (Figure 5B). The control dsRNA had no effect on *Tup1* gene expression, indicating that the *Tup1*-dsRNA has gene-targeting specificity.

### 2.5. Effect of Exogenous Application of Tup1-dsRNA on the Growth and Morphology of Fusarium oxysporum Hyphae

**Figure 6:**
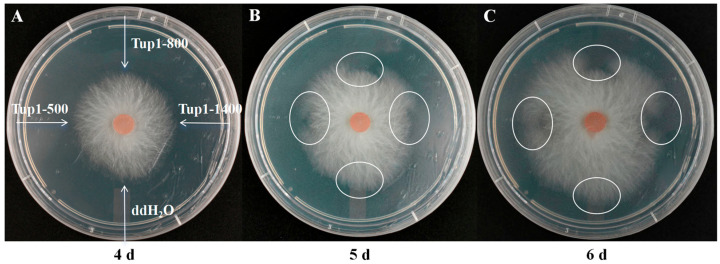
Effect of *Tup1*-dsRNA on the growth of *F. oxysporum* hyphae on plates (*n* = 3). (**A**) Application of ddH_2_O, Tup1-500, Tup1-800, and Tup1-1400 to different areas of *F. oxysporum* hyphae on the fourth day of plate growth; (**B**) Growth of *F. oxysporum* hyphae on plates on the fifth day; (**C**) Growth of *F. oxysporum* hyphae on plates on the sixth day.

To investigate the inhibitory effect of dsRNA on the growth of *F. oxysporum* hyphae, Tup1-500, Tup1-800, and Tup1-1400 were applied to *F. oxysporum* plates (*n* = 3). In our previous results, we found that the control dsRNA had no effect on the growth of *F. oxysporum*; therefore, the control dsRNA group was not included in this experiment. On the fourth day of *F. oxysporum* growth on the plates, ddH_2_O, Tup1-500, Tup1-800, and Tup1-1400 were separately applied to different areas at the growing edge of the hyphae (Figure 6A). On the fifth day, it was observed that hyphal growth was inhibited to varying degrees in all areas except where ddH_2_O had been applied (Figure 6B), and this inhibition persisted on the sixth day (Figure 6C).

**Figure 7:**
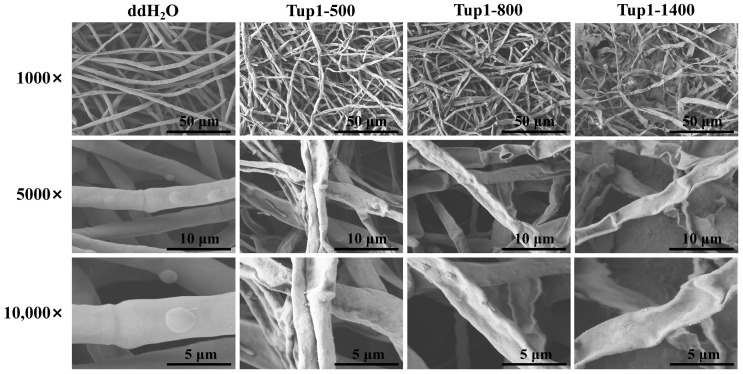
Electron micrographs of *F. oxysporum* hyphae after *Tup1*-dsRNA treatment (Magnifications: 1000×, 5000×, 10,000×, scale bars shown in the images).

To further observe the effect of *Tup1*-dsRNA on the morphology of *F. oxysporum* hyphae, the hyphae at the edges of the plates treated with ddH_2_O, Tup1-500, Tup1-800, and Tup1-1400 were examined under an electron microscope. Compared to the control group, varying degrees of shrinkage and a rougher surface were exhibited by the hyphae treated with the three different lengths of *Tup1*-dsRNA. The most severe shrinkage was observed in the hyphae treated with Tup1-1400 (Figure 7). These results indicate that *Tup1*-dsRNA can inhibit the growth of *F. oxysporum* hyphae and affect its morphology.

### 2.6. Exogenous Application of Tup1-dsRNA Alleviates the Occurrence of Atractylodes macrocephala Root Rot

**Figure 8:**
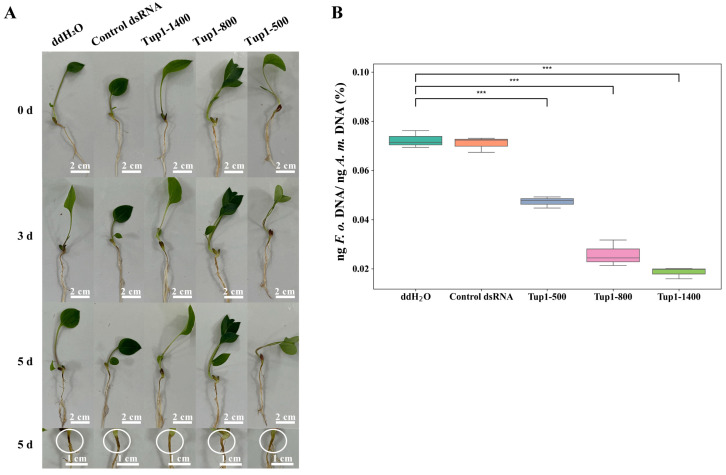
*Tup1*-dsRNA can reduce the infection of *F. oxysporum***.** (**A**) After 24 h of *Tup1*-dsRNA treatment at the wound site on the root of *A. macrocephala*, the severity of lesions caused by *F. oxysporum* infection was reduced (indicated by white circles in the figure) (*n* = 6). (**B**) *Tup1*-dsRNA inhibits the colonization ability of *F. oxysporum* in the roots of *A. macrocephala*. *** marks *p* < 0.001.

We explored whether *Tup1*-dsRNA could be used as a new and potentially effective agent for controlling plant diseases, and *Tup1*-dsRNA was applied to the roots of *A. macrocephala* to evaluate its effect on controlling root rot (*n* = 6). To determine whether dsRNA itself affects the growth of *A. macrocephala* roots, we also included a control dsRNA group. First, a cut was made on the root of *A. macrocephala* at the two-leaf stage and ddH_2_O, control dsRNA, Tup1-500, Tup1-800, and Tup1-1400 were applied, respectively. After being placed in a light incubator for 24 h, the roots were inoculated with *F. oxysporum* spore suspension (1 × 10^6^ cfu mL^−1^). Then, 5 days after inoculation with *F. oxysporum*, symptoms of rot were observed on the roots treated with ddH_2_O and control dsRNA, while reduced symptoms of the disease were observed on the roots treated with *Tup1*-dsRNA (Figure 8A). Subsequently, we conducted RT-qPCR analysis on the roots of *A. macrocephala* 5 days after treatment to detect the colonization of *F. oxysporum*. The results showed that the colonization of *F. oxysporum* was significantly lower in all groups treated with *Tup1*-dsRNA compared to the ddH_2_O and control dsRNA groups (Figure 8B) (*p* < 0.001). These findings suggest that all three different lengths of *Tup1*-dsRNA effectively reduce the colonization of *F. oxysporum* in *A. macrocephala* roots, thereby significantly alleviating root rot symptoms.

**Figure 9:**
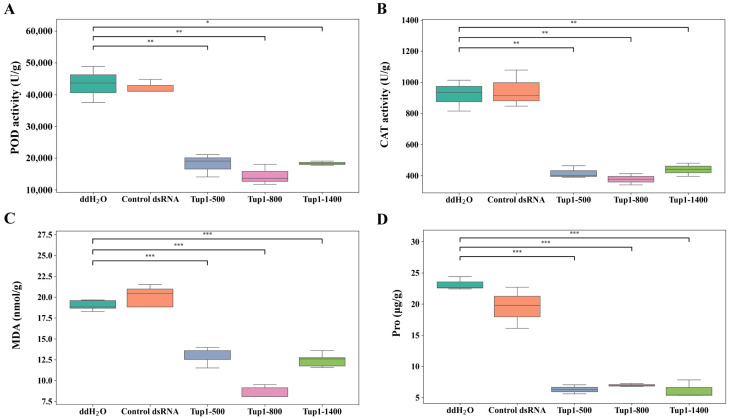
Analysis of POD and CAT enzyme activities, MDA, and Pro contents in the roots of *A. macrocephala* after treatment with ddH_2_O, control dsRNA, Tup1-500, Tup1-800, and Tup1-1400 (*n* = 3). (**A**) POD activity; (**B**) CAT activity; (**C**) MDA content; (**D**) Pro content. *** marks *p* < 0.001, ** marks *p* < 0.01, * marks *p* < 0.05.

Further analysis was conducted on the fifth day of treatment to examine the activities of peroxidase (POD) and catalase (CAT), as well as the levels of malondialdehyde (MDA) and proline (Pro) in the roots of *A. macrocephala*. The results showed that POD and CAT activities were higher in root tissues treated with ddH_2_O and control dsRNA, while these enzyme activities were significantly reduced following *Tup1*-dsRNA treatment. Since POD and CAT are key enzymes involved in scavenging reactive oxygen species as part of the plant’s defense response [27], this reduction suggests that *Tup1*-dsRNA may decrease the pathogenicity of *F. oxysporum*, thereby reducing the plant’s defensive response (Figure 9A,B). Moreover, MDA content, an indicator of lipid peroxidation and cell membrane oxidative damage [28], was higher in the ddH_2_O and control dsRNA groups but significantly decreased after *Tup1*-dsRNA treatment, indicating reduced oxidative stress due to decreased pathogen activity (Figure 9C). Similarly, Pro, which typically accumulates in response to stress, was also higher in the ddH_2_O and control dsRNA groups but significantly decreased following *Tup1*-dsRNA treatment (Figure 9D). Pro plays a key role in plant stress tolerance, and its levels can reflect the plant’s response to environmental stresses [29]. These findings suggest that *Tup1*-dsRNA reduces the pathogenicity of *F. oxysporum*, thereby modulating the defensive responses of *A. macrocephala* and effectively controlling root rot.

## 3. Discussion

Exogenous dsRNA-induced gene silencing represents a promising strategy for plant protection that has shown great potential and could serve as an environmentally friendly alternative to conventional fungicide treatments [30,31]. Our research demonstrated that exogenous dsRNA-induced gene-silencing technology significantly reduces the pathogenicity of *F. oxysporum*.

Different lengths of *Tup1*-dsRNA were first synthesized, and their inhibitory effect on the growth of *F. oxysporum* in vitro was validated through exogenous application. The results showed that *Tup1*-dsRNA was effectively taken up by *F. oxysporum*, inducing gene silencing and significantly reducing its growth and pathogenicity. Additionally, experiments with fluorescently labeled dsRNA confirmed that *F. oxysporum* could non-selectively uptake dsRNA from the environment. Furthermore, the application of *Tup1*-dsRNA in controlling *A. macrocephala* root rot was explored. The experimental results indicated that the symptoms of the disease were significantly alleviated in *A. macrocephala* roots treated with *Tup1*-dsRNA after inoculation with *F. oxysporum*. This finding provides a theoretical basis for developing new RNAi-based methods for plant disease control.

Although our dsRNA formulation demonstrated the ability to inhibit the virulence of *F. oxysporum*, many challenges remain for further field trials. The first challenge is production. This study was conducted in the laboratory using kits to synthesize dsRNA in vitro, with each reaction producing only microgram levels of dsRNA, which is insufficient for large-scale field application. The current research has focused on expressing dsRNA in *Escherichia coli* and directly extracting dsRNA from the bacteria, achieving milligram levels [32]. Another challenge is the instability of dsRNA in soil, which makes direct application ineffective for protecting plants from pathogen infection [33]. A promising approach to significantly enhance the stability of dsRNA involves encapsulating it in liposomes or adsorbing it onto carriers [34]. Additionally, mixing dsRNA with other compounds can enhance the functionality of dsRNA [35].

We aimed to explore the feasibility of using SIGS to control *A. macrocephala* root rot, and thus only *Tup1* was selected as the model gene for the study. *Tup1* was chosen for silencing because it acts as a virulence factor and regulates the transcriptional expression of other virulence genes [36]. Currently, there is limited research on the *F. oxysporum Tup1* gene, which adds novelty to this study and advances research on *Tup1* as a representative virulence gene of *F. oxysporum*. Both the phylogenetic analysis and amino acid sequence alignment indicated that the Tup1 protein is conserved within the *Fusarium* species, providing the potential for developing broad-spectrum dsRNA formulations to control root rot diseases in other medicinal plants. When designing dsRNA, it was found that Tup1-500, Tup1-800, and Tup1-1400 all inhibited the pathogenicity of *F. oxysporum*. During our investigations, we observed that although the inhibitory effect of Tup1-500 dsRNA on *Tup1* gene expression was not as significant as that of Tup1-800 and Tup1-1400, its impact on fungal growth and virulence could still be observed. This may be due to the fact that partial silencing of the *Tup1* gene is sufficient to affect the phenotype of *F. oxysporum*. Additionally, the partial expression of the *Tup1* gene may lead to secondary effects by regulating other metabolic pathways, thereby influencing the virulence of the fungus. Therefore, even if the inhibition of *Tup1* gene expression is not significant, it can still have a notable impact on the biological function of the fungus. However, in practical production, longer dsRNA requires a longer template, which increases the difficulty of synthesis. Therefore, the length of the dsRNA fragment is not necessarily better the longer it is. Genes such as *Fub*, which encodes fusaric acid [37], and *Sge*, which is essential for full virulence [38], are worthy targets for designing targeted silencing of virulence genes. More virulence genes are expected to be discovered in future research.

After targeting the *Tup1* gene with *Tup1*-dsRNA, the growth and colonization ability of *F. oxysporum* were affected. Comparing the inhibitory effect of dsRNA in vitro with that on *A. macrocephala* roots, it was found that after 24 h of dsRNA pre-treatment at the wound site, the colonization ability of *F. oxysporum* was still inhibited at 5 days after inoculation. This may be partly due to the non-specific action of siRNA on similar fragments within *A. macrocephala*, mediating the production of secondary siRNA [39], thereby amplifying the inhibition of *F. oxysporum*.

In conclusion, exogenous dsRNA-induced gene-silencing technology demonstrated significant effectiveness in controlling *A. macrocephala* root rot caused by *F. oxysporum*, showcasing its potential as a novel biological fungicide. However, challenges related to dsRNA production and stability need to be addressed to achieve widespread field application. Additionally, further research on other virulence gene targets will help develop more efficient and broad-spectrum RNAi fungicides.

## 4. Materials and Methods

### 4.1. Fungal Strains and Culture Conditions

We obtained *F. oxysporum* from Beina Chuanglian Biotech Institute (Chaoyang District, Beijing, China) and cultured T7 on potato dextrose agar (PDA) plates at 25 °C. To prepare the medium, we used 0.7 g of potato dextrose broth (PDB) (Hangzhou Microbial Reagent, Hangzhou, China) and 0.375 g of agar (Sangon Biotech, Shanghai, China) per plate. Conidial suspensions were generated from 5- to 6-day-old PDB cultures. Using a sterile punch, we extracted fungal plugs and inoculated them into 100 mL of PDB liquid medium. We then filtered the conidial suspensions through sterile gauze (Solarbio, Beijing, China) and determined the concentration of conidia using a hemocytometer (Hirschmann, Eberstadt, Germany), adjusting it to the desired concentration [40].

### 4.2. Plant Materials

*A. macrocephala* seeds were obtained from the Traditional Chinese Medicine Research Institute (Jinhua, Zhejiang, China). The seeds underwent a pre-treatment process that included soaking for 12 h, followed by germination on moistened blotting paper at 25 ± 2 °C for 15 days. Seedlings, upon reaching the first leaf stage, were transplanted into sterile soil and grown in an artificial climate chamber (Boxun, Shanghai, China). Conditions within the chamber were maintained at 25 ± 2 °C, with a light intensity of 1600 lux, a 16/8 h light/dark cycle, and 75% humidity until the seedlings developed to the two-leaf stage.

### 4.3. Preparation of Fusarium oxysporum Protoplasm

We cultured *F. oxysporum* in 100 mL of Yeast Extract Peptone Dextrose (YPD) (Hangzhou Microbial Reagent, Hangzhou, China) liquid medium at 25 °C with shaking at 200 rpm for 3 days. The fungal suspension was filtered through sterile gauze to remove mycelia, followed by centrifugation at 3000 rpm for 5 min. After discarding the supernatant, we resuspended the collected spores in sterile distilled water and adjusted their concentration to 3 × 10^8^ cfu mL^−1^ using a hemocytometer. Subsequently, 1 mL of this spore suspension was inoculated into 100 mL of YPD medium containing 100 μg mL^−1^ ampicillin (Sigma-Aldrich, St. Louis, MO, USA) and cultured overnight under the same conditions. We then harvested and washed the mycelia sequentially with sterile distilled water and 1.2 M KCl (Sigma-Aldrich, St. Louis, MO, USA). The mycelia were then digested in an enzyme solution containing 500 mg Driselase (GlpBio, Montclair, CA, USA), 200 mg Lysing Enzymes (GlpBio, Montclair, CA, USA), and 200 mg Snailase (GlpBio, Montclair, CA, USA) in 20 mL of 1.2 M KCl [41], with shaking at 28 °C and 90 rpm for 2–3 h. Finally, we isolated the resulting protoplasts by filtration and centrifugation at 5000 rpm for 90 s at 4 °C, washing them three times with ice-cold Sucrose–tris–calcium (STC) buffer. A total of 100 mL of STC buffer contains of 1 mL 1.2 M Sorbitol (Aladdin, Shanghai, China), 1 mL 1 M Tris-HCl (pH 7.5) (Solarbio, Beijing, China), 2 mL 2.5 M CaCl_2_ (Aladdin, Shanghai, China), and 96 mL ddH_2_O.

### 4.4. DNA Extraction and Tup1 Gene Amplification

We extracted genomic DNA from 20 mg of *F. oxysporum* mycelium using the Rapid Fungi Genomic DNA Isolation Kit (Sangon Biotech, Shanghai, China). Specific primers were designed to amplify the *Tup1* gene (Table 1), with PCR conditions set at 94 °C for 3 min for initial denaturation, followed by 35 cycles of 94 °C for 15 s, 55 °C for 20 s, and 72 °C for 2 min, with a final extension at 72 °C for 10 min. The amplified sequences were confirmed via agarose gel electrophoresis and analyzed using BLAST (blast.ncbi.nlm.nih.gov/Blast.cgi, Date of access: 16 March 2024), CD SERVER (www.ncbi.nlm.nih.gov/Structure/cdd/wrpsb.cgi, Date of access: 16 March 2024), MEGA 11 (www.megasoftware.net, Date of access: 17 March 2024), and Clustalw (http://www.clustal.org, Date of access: 17 March 2024) [42,43].

### 4.5. Total RNA Extraction and cDNA Synthesis

Total RNA was extracted from *F. oxysporum* using TRIzol Reagent (Thermo Fisher, Waltham, MA, USA), following the same grinding method used for DNA extraction. We then performed reverse transcription using the M-MuLV First Strand cDNA Synthesis Kit (Sangon Biotech, Shanghai, China) according to the manufacturer’s instructions, with 0.5 µg of total RNA serving as the template.

### 4.6. dsRNA Synthesis

Three different lengths of *Tup1*-dsRNA were designed: 500 bp, 800 bp, and 1400 bp (referred to as Tup1-500, Tup1-800, and Tup1-1400). Primer pairs for *Tup1*-dsRNA were designed with T7 promoter sequences at the ends of both the forward and reverse primers for the synthesis of dsRNA templates (Table 2) [44]. Using *F. oxysporum* cDNA and the designed primers, we synthesized dsRNA templates under the following PCR conditions: initial denaturation at 94 °C for 3 min, followed by 35 cycles of 94 °C for 45 s, 50 °C for 45 s, and 72 °C for 60 s, with a final extension at 72 °C for 5 min. The dsRNAs were generated using the T7 RNAi Transcription Kit (Vazyme, Nanjing, China). The control dsRNA was synthesized using the control template provided in the kit. The synthesized dsRNA was then purified using the magnetic bead purification method recommended by the kit.

### 4.7. Assessment of dsRNA Uptake by Fusarium oxysporum

During the in vitro synthesis of fluorescein-labeled dsRNA using the T7 RNAi Transcription Kit (Vazyme, Nanjing, China), we replaced the regular Nucleoside triphosphate (NTP) mixture in the kit with fluorescein-labeled NTP. The dsRNA was incubated with *F. oxysporum* spores for 24 h at 25 °C. We observed the uptake of dsRNA by *F. oxysporum* using a fluorescence-inverted microscope (Carl Zeiss AG, Oberkochen, Germany). Additionally, *F. oxysporum* protoplasts were observed under the same microscope to assess dsRNA uptake. 

### 4.8. Effects of Tup1-dsRNA on the Growth of Fusarium oxysporum Conidia In Vitro

Conidial growth was studied using sterile 96-well plates (SAINING, Suzhou, China) to evaluate the effects of *Tup1*-dsRNA [18,19]. Equal volumes of a spore suspension at a concentration of 1 × 10^6^ cfu mL^−1^ and *Tup1*-dsRNA (final concentration 50 ng μL^−1^) were added to the wells (each treatment *n* = 6, total assay volume 200 μL). The controls were prepared in PDB and included the following components: (i) ddH_2_O + *F. oxysporum* conidia, (ii) control dsRNA + *F. oxysporum* conidia. The plates were incubated at 25 °C, and fungal growth was assessed by measuring the optical density at OD_595_ using a microplate reader spectrophotometer (Bio-Rad, Cressier, Switzerland) at various time points between 0 and 24 h. After incubation, we collected the *F. oxysporum* mycelium, observed its growth under an optical microscope (Sunny Optical Technology, Yuyao, China), and then washed it with ddH_2_O by centrifugation. Fungal transcript analysis was performed to evaluate the silencing of the *Tup1* gene.

### 4.9. Effects of Tup1-dsRNA on Fusarium oxysporum Mycelial Morphology and Growth In Vitro

We used a sterile puncher to create holes in the *F. oxysporum* PDA plate and then inoculated the fungi into the center of a new PDA plate covered with cellophane (Acmec, Shanghai, China). After incubation at 25 °C for 4 days, ddH_2_O and *Tup1*-dsRNA (at a concentration of 100 ng μL^−1^) were added around the edges of the hyphal growth. Incubation continued until the sixth day. Then, the hyphal edges were cut, made into slices, and observed under an electron microscope to examine the hyphal morphology. During the observation, we performed a morphological analysis using a scanning electron microscope (SEM) (Carl Zeiss AG, Oberkochen, Germany) at magnifications of 1000×, 5000×, and 10,000× to conduct a detailed examination of the hyphal morphology, allowing us to evaluate the effects of *Tup1*-dsRNA treatment on the morphology of *F. oxysporum* hyphae.

### 4.10. Application of Tup1-dsRNA on the Roots of Atractylodes macrocephala

*A. macrocephala* seedlings at the two-leaf stage were transferred to trays with moistened blotting paper. A 5 mm wound was made on the root 5 mm below the stem using a dissecting needle. We applied *Tup1*-dsRNA (5 µL of 100 ng µL^−1^) to the wound, with control dsRNA and ddH_2_O serving as controls. The trays were placed in an artificial climate chamber (25 °C, 1600 lux light intensity, 16 h light/8 h dark) for 24 h. Following this, the wound was inoculated with 5 µL of *F. oxysporum* PDB culture homogenate. The seedlings were incubated for 5 days, and symptoms were observed. To detect the colonization of *F. oxysporum* in the roots of *A. macrocephala*, root tissues were collected on the fifth day after treatment. Total DNA was extracted from the plant roots using the Rapid Plant Genomic DNA Isolation Kit (Sangon Biotech, Shanghai, China) and used as a template. RT-qPCR analysis was performed using specific primer pairs (Table 3). The DNA content of *A. macrocephala* and *F. oxysporum* was quantified using standard curves to determine the colonization of *F. oxysporum*.

### 4.11. Biochemical Indicators and Enzyme Activity Assays

To measure the activities of POD and CAT, as well as the contents of MDA and Pro in the roots of *A. macrocephala*, we used the appropriate detection kits (POD: #BC0095; CAT: #BC0205; MDA: #BC0025; Pro: #BC0295) (Solarbio, Beijing, China) and followed the manufacturer’s instructions. For each indicator, 0.1 g of *A. macrocephala* root tissue was used for the measurements, with three biological replicates.

### 4.12. Gene Expression Analysis by Reverse Transcription Quantitative Polymerase Chain Reaction

The mRNA expression analysis was conducted using RT-qPCR to evaluate the silencing of pathogenic genes. A total of 1 μL of cDNA was used as a template for RT-qPCR in a real-time PCR system (Thermo Fisher, Waltham, MA, USA); the primers used are shown in Table 4. Amplification was performed in a 10 μL reaction mixture containing 5 µL SYBR^®^ Green and 5 pmol oligonucleotides. Each sample had three biological replicates. We used primers specifically designed to study the expression of the *Tup1* pathogenic gene, and the PCR conditions were set as follows: 95 °C for 5 min, followed by 35 cycles of 95 °C for 15 s and 60 °C for 60 s.

### 4.13. Statistical Analysis

Quantitative PCR data were transformed using the 2^−ΔΔCT^ method [45]. Statistical analysis was performed using SPSS 20.0 (IBM Corp., Armonk, NY, USA). One-way ANOVA was used for comparisons between groups [46]. Significant differences between different treatment groups are marked in the figure. GraphPad 9.5.0 (GraphPad Software, San Diego, CA, USA) was used for plotting.

## Figures and Tables

**Table 1:** Primers for amplifying the *Tup1* gene.

Gene	5′-3′ Sequence	Annealing Temperature
*Tup1*	ATGTCGATGTATCCGCATCGC	63.1 °C
	TGACTTCGACAGAACCGGAC	58.0 °C

**Table 2:** Primers for amplifying the fragments of *Tup1*-dsRNA templates.

dsRNA	5′-3′ Sequence	Annealing Temperature
Tup1-500	TAATACGACTCACTATAGGGCTCAAGTCAGCCATCCGACTCC	80.8 °C
	TAATACGACTCACTATAGGGGCGAGTAAATATCTTGCTCGTGACC	79.6 °C
Tup1-800	TAATACGACTCACTATAGGGCCTTACTCTCAGAACTATCCTCCGG	78.8 °C
	TAATACGACTCACTATAGGGGACGCCATCCTCTATTGTCAGAG	79.0 °C
Tup1-1400	TAATACGACTCACTATAGGGTCTCGTTCCTCCTCAGCCAGG	79.9 °C
	TAATACGACTCACTATAGGGGAGCCAGTGGCAAGTATCTTCC	79.0 °C

**Table 3:** Primers used for RT-qPCR analysis of *F. oxysporum* colonization in the roots of *A. macrocephala*.

Primer	5′-3′ Sequence	Annealing Temperature
Q-*Matk* (*A. macrocephala*)	TGCCCCAATGCGTTACAAAATTTCG	70.7 °C
	AAAGCCTTCAATGGTCCGCAGTC	66.6 °C
Q-*Prot* (*F. oxysporum*)	CGAGGTGTGGATTTAGGAGATG	58.9 °C
	GTGCTGATTCGTTGTACGTTATTC	58.8 °C

**Table 4:** Primers Used for Reverse Transcription Quantitative Polymerase Chain Reaction (RT-qPCR) Analysis of *Tup1* Gene Expression.

Primer	5′-3′ Sequence	Annealing Temperature
Q-*FoTup1*	GCCTCAAGCAGACGTATGAAGATG	63.1 °C
	TTGCCAGGAGCGATAGAAGGAG	63.5 °C
Q-*Actin*	GAGGGACCGCTCTCGTCGT	62.1 °C
	GGAGATCCAGACTGCCGCTGAG	65.8 °C

## Data Availability

Data are contained within the article.

## Abbreviations

*F. oxysporum*	*Fusarium oxysporum*
*A. macrocephala*	*Atractylodes macrocephala*
dsRNA	Double-stranded RNA
siRNA	Small interfering RNA
OD	Optical density
TCA	Tricarboxylic acid (cycle)
PDB	Potato dextrose broth
PDA	Potato dextrose agar
YPD	Yeast peptone dextrose
SIGS	Spray-induced gene silencing
SEM	Scanning electron microscope
NTP	Nucleoside triphosphate
STC	Sucrose–tris–calcium buffer
POD	Peroxidase
CAT	Catalase
MDA	Malondialdehyde
Pro	proline
